# Supplementary material for: Modeling glioblastoma heterogeneity as a dynamic network of cell states
Source: Mol Syst Biol. 2021 Sep 16;17(9):e10105. doi: 10.15252/msb.202010105 (PMC8444284; doi:10.15252/msb.202010105)
Supplement: Supplementary file 6 — Source Data for Figure 5 [file MSB-17-e10105-s004.zip › Figure5A_sourcedata/GSEA_3017/nosigdb_stateB.GseaPreranked.1622795021725/RICHARDS_NOWAKOWSKI_SCIENCE_2017_IPC_NEN1_UPREG.html]

Details for gene set RICHARDS\_NOWAKOWSKI\_SCIENCE\_2017\_IPC\_NEN1\_UPREG[GSEA]

|  || Dataset | state43017 |
| Phenotype | NoPhenotypeAvailable |
| Upregulated in class | na\_neg |
| GeneSet | RICHARDS\_NOWAKOWSKI\_SCIENCE\_2017\_IPC\_NEN1\_UPREG |
| Enrichment Score (ES) | -0.42650074 |
| Normalized Enrichment Score (NES) | -2.5515013 |
| Nominal p-value | 0.0 |
| FDR q-value | 0.0021310542 |
| FWER p-Value | 0.003 |
Table: GSEA Results Summary

  

Fig 1: Enrichment plot: RICHARDS\_NOWAKOWSKI\_SCIENCE\_2017\_IPC\_NEN1\_UPREG      
 Profile of the Running ES Score & Positions of GeneSet Members on the Rank Ordered List

  

| PROBE | GENE SYMBOL | GENE\_TITLE | RANK IN GENE LIST | RANK METRIC SCORE | RUNNING ES | CORE ENRICHMENT || 1 | PDE1C |  |  | 166 | 0.445 | -0.1710 | No |
| 2 | SYNE2 |  |  | 203 | 0.413 | -0.1693 | No |
| 3 | CCSAP |  |  | 249 | 0.379 | -0.1839 | No |
| 4 | MACF1 |  |  | 342 | 0.328 | -0.2686 | No |
| 5 | SOGA1 |  |  | 378 | 0.314 | -0.2776 | No |
| 6 | EZR |  |  | 403 | 0.307 | -0.2725 | No |
| 7 | MEIS2 |  |  | 405 | 0.307 | -0.2363 | No |
| 8 | NR2F1 |  |  | 473 | 0.287 | -0.2922 | No |
| 9 | COTL1 |  |  | 499 | 0.281 | -0.2916 | No |
| 10 | NFIC |  |  | 531 | 0.276 | -0.2999 | No |
| 11 | PAX6 |  |  | 617 | 0.261 | -0.3834 | No |
| 12 | HEG1 |  |  | 639 | 0.257 | -0.3805 | No |
| 13 | TSPAN5 |  |  | 646 | 0.256 | -0.3572 | No |
| 14 | CA12 |  |  | 698 | -0.274 | -0.3929 | Yes |
| 15 | FLRT3 |  |  | 699 | -0.275 | -0.3592 | Yes |
| 16 | CHD7 |  |  | 706 | -0.284 | -0.3325 | Yes |
| 17 | MMP16 |  |  | 707 | -0.285 | -0.2976 | Yes |
| 18 | MYO10 |  |  | 710 | -0.305 | -0.2628 | Yes |
| 19 | SOX4 |  |  | 712 | -0.305 | -0.2267 | Yes |
| 20 | ZEB1 |  |  | 717 | -0.317 | -0.1932 | Yes |
| 21 | PRDX1 |  |  | 719 | -0.326 | -0.1547 | Yes |
| 22 | HES6 |  |  | 752 | -0.523 | -0.1340 | Yes |
| 23 | DANCR |  |  | 753 | -0.572 | -0.0638 | Yes |
| 24 | SOX11 |  |  | 754 | -0.576 | 0.0068 | Yes |
Table: GSEA details [plain text format]

  

Fig 2: RICHARDS\_NOWAKOWSKI\_SCIENCE\_2017\_IPC\_NEN1\_UPREG: Random ES distribution      
 Gene set null distribution of ES for **RICHARDS\_NOWAKOWSKI\_SCIENCE\_2017\_IPC\_NEN1\_UPREG**
